# Inhibitory Effects of Benzaldehyde Derivatives from the Marine Fungus *Eurotium* sp. SF-5989 on Inflammatory Mediators via the Induction of Heme Oxygenase-1 in Lipopolysaccharide-Stimulated RAW264.7 Macrophages

**DOI:** 10.3390/ijms151223749

**Published:** 2014-12-19

**Authors:** Kyoung-Su Kim, Xiang Cui, Dong-Sung Lee, Wonmin Ko, Jae Hak Sohn, Joung Han Yim, Ren-Bo An, Youn-Chul Kim, Hyuncheol Oh

**Affiliations:** 1 College of Pharmacy, Wonkwang University, Iksan 570-749, Korea; E-Mails: pipo5@wku.ac.kr (K.-S.K.); loodia@wku.ac.kr (X.C.); rabis815@naver.com (W.K.); 2 Professional Graduate School of Oriental Medicine, Wonkwang University, Iksan 570-749, Korea; 3 Key Laboratory of Natural Resources and Functional Molecules of the Changbai Mountain, Affiliated Ministry of Education, Yanbian University College of Pharmacy, 977 Gongyuan Road, Yanji 133002, China; E-Mail: anrb@ybu.edu.cn; 4 Inha Research Institute for Medical Sciences, Inha University School of Medicine, Incheon 400-712, Korea; E-Mail: dongsunglee@inha.ac.kr; 5 College of Medical and Life Sciences, Silla University, Busan 617-736, Korea; E-Mail: jhsohn@silla.ac.kr; 6 Korea Polar Research Institute, KORDI, 7-50 Songdo-dong, Yeonsu-gu, Incheon 406-840, Korea; E-Mail: jhyim@kopri.re.kr

**Keywords:** benzaldehyde derivatives, marine fungus, *Eurotium rubrum*, RAW264.7 macrophages, heme oxygenase-1, anti-inflammatory effect, nuclear factor-κB

## Abstract

Two benzaldehyde derivatives, flavoglaucin (**1**) and isotetrahydro-auroglaucin (**2**), were isolated from the marine fungus *Eurotium* sp. SF-5989 through bioassay- and ^1^H NMR-guided investigation. In this study, we evaluated the anti-inflammatory effects of these compounds in lipopolysaccharide (LPS)-stimulated RAW264.7 macrophages. We demonstrated that compounds **1** and **2** markedly inhibited LPS-induced nitric oxide (NO) and prostaglandin E2 (PGE_2_) production by suppressing inducible nitric oxide synthase (iNOS) and cyclooxygenase-2 (COX-2) protein expression without affecting cell viability. We also demonstrated that the compounds reduced the secretion of pro-inflammatory cytokines such as tumor necrosis factor-α (TNF-α), interleukin-1β (IL-1β) and interleukin-6 (IL-6). Furthermore, compounds **1** and **2** inhibited LPS-induced nuclear factor-κB (NF-κB) activation by suppressing phosphorylation of IkappaB (IκB). These results indicated that the anti-inflammatory effects of these benzaldehyde derivatives in LPS-stimulated RAW264.7 macrophages were due to the inactivation of the NF-κB pathway. In addition, compounds **1** and **2** induced heme oxygenase-1 (HO-1) expression through the nuclear transcription factor-E2–related factor 2 (Nrf2) translocation. The inhibitory effects of compounds **1** and **2** on the production of pro-inflammatory mediators and on NF-κB binding activity were reversed by HO-1 inhibitor tin protoporphyrin (SnPP). Thus, the anti-inflammatory effects of compounds **1** and **2** also correlated with their ability of inducing HO-1 expression.

## 1. Introduction

Macrophages induced by lipopolysaccharide (LPS) produce a variety of inflammatory cytokines including tumor necrosis factor-α (TNF-α), interleukin-1β (IL-1β), interleukin-6 (IL-6) and other inflammatory mediators [1,2]. In many inflammatory conditions, the pro-inflammatory and cytotoxic mediators including nitric oxide (NO) and prostaglandins (PGs) are released through the activity of their inducible enzymes, such as inducible nitric oxide synthase (iNOS) and cyclooxygenase-2 (COX-2) [3,4,5,6]. The iNOS and COX systems are often present together, share a number of similarities, and play fundamental roles in similar pathophysiological inflammatory conditions [3,4,5,6]. Accordingly, inhibition of the production of these inflammatory mediators might be useful for the treatment of various inflammatory diseases. Heme oxygenase-1 (HO-1) is an inducible and rate-limiting enzyme involved in the catabolism of heme, a process that leads to the formation of biliverdin, ferrous iron, and carbon monoxide [7]. HO-1 is known as a critical molecule for regulating inflammatory responses. The by-products of HO-1 inhibit the production of pro-inflammatory cytokines such as TNF-α, IL-1β, and IL-6 in activated macrophages [8,9,10]. Gene expression of HO-1 is modulated by nuclear transcription factor-E2 related factor 2 (Nrf2), and under normal conditions, Nrf2 is combined with Kelch-like ECH-associated protein (Keap1) in the cytosol of cells [11]. However, under stressful conditions, Nrf2 separates from Keap1, and translocates into the nucleus, where it activates transcription of antioxidant genes through binding to the antioxidant response element (ARE). The anti-inflammatory action of HO-1 is mediated by inhibiting the production of pro-inflammatory mediators through nuclear factor-κB (NF-κB) inactivation [12]. NF-κB activation is involved in immune and inflammatory responses [13]. Nuclear translocation of NF-κB by various stimuli including TNF-α and LPS triggers the expression of inflammatory genes. As such, it has been regarded as a valuable target in the development of agents for inflammation-related diseases [14].

Marine microorganisms including bacteria, cyanobacteria, microalgae and fungi have become an important source of new and pharmacologically active secondary metabolites [15]. In particular, marine fungi have attracted much interest as promising potential sources of novel antiviral, antibacterial, anti-inflammatory, and anticancer agents [16]. In our previous study, we isolated two natural diketopiperazine-type indole alkaloids, neoechinulins A and B, which demonstrated anti-inflammatory effects [17]. In our continuous search for anti-inflammatory secondary metabolites, two benzaldehyde derivatives flavoglaucin (**1**) and isotetrahydro-auroglaucin (**2**) were additionally isolated from the extracts of marine fungus *Eurotium* sp. SF-5989 cultures. In the present study, we describe the inhibitory effects and underlying mechanism of **1** and **2** on the inflammatory process in LPS-stimulated RAW264.7 macrophages.

## 2. Results and Discussion

### 2.1. Identification of Flavoglaucin (**1**) and Isotetrahydro-Auroglaucin (**2**), and Their Effects on the Viability of RAW264.7 Macrophages

Two metabolites were isolated from the organic extract of the culture broth of the marine-derived fungi *Eurotium* sp. SF-5989 by application of various chromatographic methods. The structures were fully characterized on the basis of analysis of NMR and MS data in comparison with literature values [18,19]. The purities of compounds **1** and **2** were estimated to be approximately more than 94.0% and 85.7%, respectively, by HPLC analysis. In addition, comparison of HPLC chroamtograms and ^1^H NMR spectra for each compound suggested that compound **2** existed as a major impurity in purified compound **1**, and *vice versa*.

**Figure 1:**
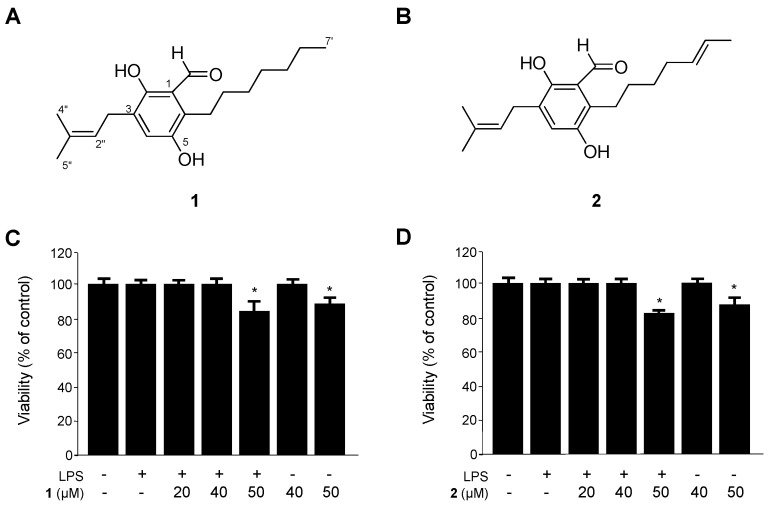
Chemical structures of **1** and **2** (**A**,**B**) from *Eurotium* sp. SF-5989, and the effects on cell viability (**C**,**D**) of RAW264.7 macrophages. RAW264.7 macrophages were incubated for 48 h with the indicated concentrations of **1** and **2**, or pretreated with the indicated concentrations of compounds **1** and **2** for 12 h, and then stimulated with LPS (1 μg/mL) for 18 h. Data are represented as the mean ± standard deviation (S.D.) of three independent experiments. * *p* < 0.05 *vs.* control.

Firstly, we evaluated the cytotoxicity of **1** and **2** on RAW264.7 macrophages by using an 3-[4,5-dimethylthiazol-2-yl]-2,5-diphenyltetrazolium bromide (MTT) assay. As shown in Figure 1, cell viability was not significantly altered up to 40 µM concentrations of **1** and **2**. Therefore, for all subsequent experiments, the concentration range of **1** and **2** were maintained between 5 and 40 µM. It was previously reported that this type of fungal metabolite has antioxidant activity [20,21]. In addition, flavoglaucin (**1**) was reported to possess various biological effects, including cytotoxicity [22], antitumor-promoting activity [23], human opioid receptor binding affinity [24], and protein tyrosine phosphatase 1B inhibitory effects [25]. Nevertheless, there have been no previous studies on the anti-inflammatory effects of this type of fungal metabolite. Thus, we investigated the potential anti-inflammatory effects of compounds **1** and **2** in LPS-induced RAW 264.7 macrophages. Furthermore, the mechanisms underlying the anti-inflammatory effects of compounds **1** and **2** were demonstrated for the first time.

### 2.2. Effects of **1** and **2** on HO-1 mRNA and Protein Expression via Nuclear Translocation of Nuclear Transcription Factor-E2–Related Factor 2 (Nrf2) in RAW264.7 Macrophages

**Figure 2:**
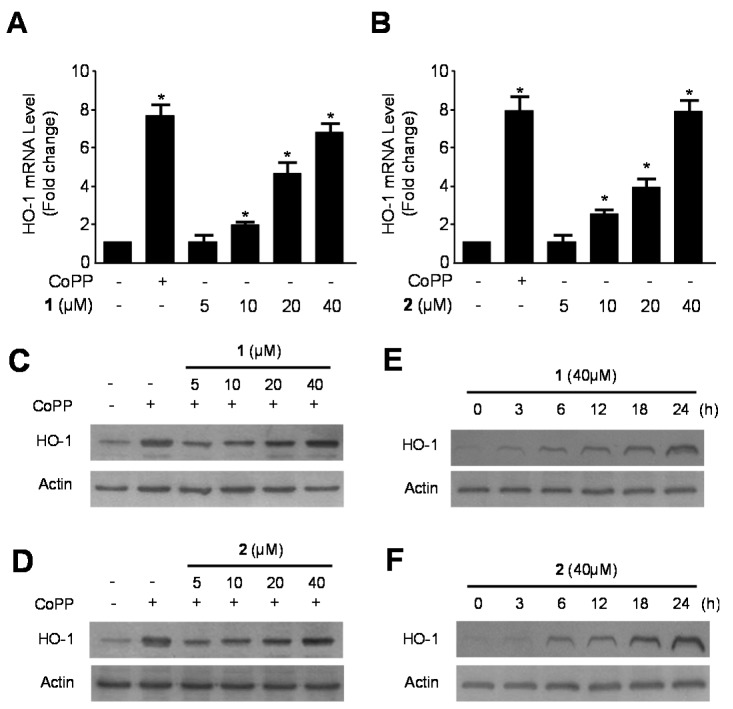
Effects of **1** and **2** on heme oxygenase-1 (HO-1) mRNA (**A**,**B**) and protein (**C**–**F**) expression in RAW264.7 macrophages. RAW264.7 macrophages were incubated with indicated concentrations of **1** and **2** for 12 h (**A**–**D**). For the time-course study, the cells were incubated with **1** and **2** (40 μM) for the indicated times (**E**,**F**). Data are represented as the mean ± S.D. of 3 independent experiments. Cobalt protoporphyrin (CoPP) (20 μM) was used as the positive control. * *p* < 0.05 *vs.* control.

**Figure 3:**
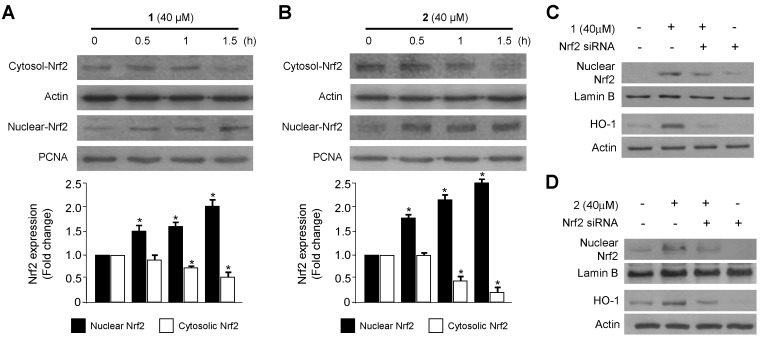
Effects of compounds **1** and **2** on the nuclear translocation of Nrf2 (**A**,**B**) and Nrf2-mediated HO-1 expression (**C**,**D**) in RAW264.7 macrophages. RAW264.7 macrophages were treated with **1** and **2** (40 μM) for 0.5, 1, and 1.5 h (**A**,**B**). RAW264.7 macrophages were transiently transfected with Nrf2 siRNA and then treated with **1** and **2** (40 μM) for 12 h (HO-1) or 1.5 h (Nuclear Nrf2) (**C**,**D**). Data are represented as the mean ± S.D. of three independent experiments. * *p* < 0.05 *vs.* control.

Along with its anti-oxidative effects, recent studies have also shown the anti-inflammatory effects of HO-1 reaction in a number of inflammatory models [26]. HO-1 and its by-products are known to have anti-oxidant, anti-inflammatory, and anti-apoptotic activities [27,28]. Nrf2, a regulator of the anti-oxidant and anti-inflammatory response, is closely linked to induction of HO-1 [29]. In recent studies, Nrf2-mediated HO-1 expression was considered as a potential therapeutic target for treating inflammatory disorders [30,31]. Therefore, we initially examined the effects of compounds **1** and **2** on HO-1 expression in RAW264.7 macrophages. Compounds **1** and **2** induced HO-1 mRNA and protein expression in a dose-dependent manner in cells treated with various concentrations of the compounds for 12 h (Figure 2A–D). The induction of HO-1 appeared 6 h into treatment with **1** and **2**, and subsequently increased in a time-dependent manner until 24 h (Figure 2E,F). Since Nrf2 plays an important role in the transcriptional activation of HO-1 gene expression [32], we investigated whether treatment with compounds **1** and **2** induced nuclear translocation of Nrf2 by western blot analysis. Cells incubated with 40 μM of **1** and **2** for 0.5, 1 and 1.5 h showed increased nuclear Nrf2 levels, and decreased cytosolic Nrf2 levels (Figure 3A,B). Furthermore, the role of Nrf2 in HO-1 expression induced by **1** and **2** were studied using siRNA against Nrf2. RAW264.7 macrophages were transiently transfected with siRNA Nrf2 and then were treated with 40 μM of compounds **1** and **2** for 12 h (HO-1) or 1.5 h (Nuclear Nrf2). As shown in Figure 3C,D, transient transfection with Nrf2 siRNA completely abolishes HO-1 expression or nuclear translocation of Nrf2 induced by **1** and **2**. These results suggest that induction of HO-1 expression by **1** and **2** occurs via the Nrf2/ARE signaling pathway in RAW264.7 macrophages.

### 2.3. Inhibitory Effects of **1** and **2** on the Production and Expression of Pro-Inflammatory Cytokines and Mediators in LPS-Stimulated RAW264.7 Macrophages

**Figure 4:**
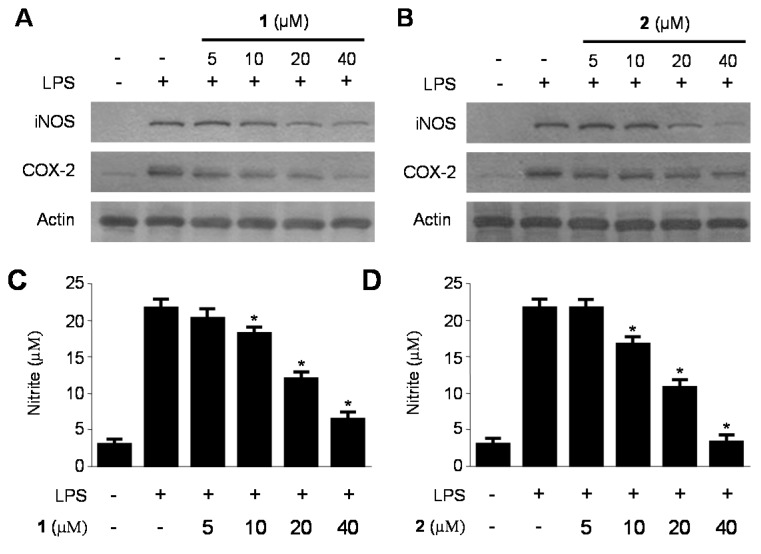
Effects of **1** and **2** on protein expression of inducible nitric oxide synthase (iNOS) and cyclooxygenase-2 (COX-2) (**A**,**B**); and on the production of nitrite (**C**,**D**) in RAW264.7 macrophages stimulated with lipopolysaccharide (LPS). RAW264.7 macrophages were pretreated with the indicated concentrations of **1** and **2** for 12 h, and then stimulated with LPS (1 μg/mL) for 18 h (**A**–**D**). Data are represented as the mean ± S.D. of three independent experiments. Western blot analysis was representative of three independent experiments with similar results. * *p* < 0.05 *vs.* LPS treatment alone.

**Figure 5:**
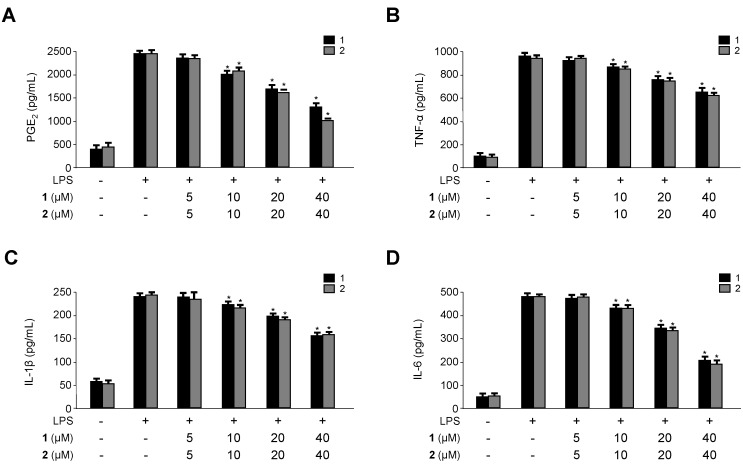
Effects of **1** and **2** on the production of prostaglandin E2 (PGE_2_), tumor necrosis factor-α (TNF-α), interleukin-1β (IL-1β), and interleukin-6 (IL-6) (**A**–**D**) in RAW264.7 macrophages stimulated with lipopolysaccharide (LPS). RAW264.7 macrophages were pretreated with the indicated concentrations of **1** and **2** for 12 h, and then stimulated with LPS (1 μg/mL) for 18 h. Data are represented as the mean ± S.D. of three independent experiments. * *p* < 0.05 *vs.* LPS treatment alone.

The inflammation response is a complex reaction of the immune system that is regulated by many inflammatory mediators, such as NO, prostaglandins, and cytokines. NO, which is generated by iNOS in activated macrophages, has physiological and pathological effects including inflammation [33,34]. Excessive NO production causes a variety of inflammatory diseases [35,36]. In addition to NO, LPS-stimulated macrophages also produce or express pro-inflammatory mediators and cytokines such as iNOS, COX-2, PGE_2_, TNF-α, IL-1β and IL-6 [37]. PGE_2_, which is a major COX-2 derived product at inflammatory sites, can trigger the development of inflammatory diseases [38]. TNF-α, IL-1β and IL-6 play an important role in inflammatory responses, and modulation of these cytokines is a major mechanism of inflammation [39]. Therefore, searching for new agents with the potential to lower NO production would be useful in the treatment of inflammatory diseases. It is well known that the expression of iNOS and COX-2, the key enzymes for NO and PGE_2_ production, is up-regulated in activated macrophages. Therefore, we confirmed the effects of **1** and **2** on the production and expression of pro-inflammatory cytokines and mediators, such as NO, PGE_2_, TNF-α, IL-6, IL-1β, iNOS, and COX-2 in LPS-stimulated RAW264.7 macrophages. RAW264.7 macrophages were treated with the indicated concentrations of **1** and **2** for 12 h prior to LPS treatment for 18 h. As shown in Figure 4 and Figure 5, compounds **1** and **2** decreased NO, PGE_2_, TNF-α, IL-6 and IL-1β production as well as iNOS and COX-2 protein expression in a dose-dependent manner. In addition, we have added the experiments on the nitrite production and protein iNOS expression in RAW264.7 macrophages stimulated with lower concentrations of LPS. As a result, we got the same pattern of results as those for 1 μg/mL of LPS-induced cell conditions, but the 10 μg/mL LPS-treated condition was a little production of NO compared with 1 μg/mL LPS-treated condition (Figure S1).

### 2.4. Effects of **1** and **2** on Nuclear Factor-κB (NF-κB) Activation in LPS-Stimulated RAW264.7 Macrophages

**Figure 6:**
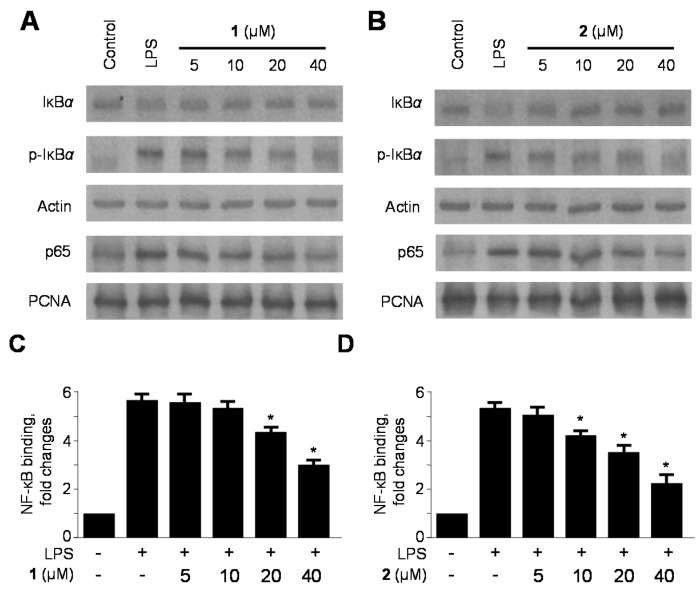
Effects of compounds **1** and **2** on nuclear factor-κB (NF-κB) activation (**A**,**B**) and NF-κB DNA-binding activity (**C**,**D**) in RAW264.7 macrophages stimulated with lipopolysaccharide (LPS). RAW264.7 macrophages were pretreated with the indicated concentrations of compounds **1** and **2** for 12 h, and then stimulated with LPS (1 μg/mL) for 30 min. Western blot analysis was representative of three independent experiments with similar results. A commercially available NF-κB enzyme-linked immunosorbent assay (ELISA) kit (Active Motif) was used to test nuclear extracts and determine the degree of NF-κB binding. Data are represented as the mean ± S.D. of three independent experiments. * *p* < 0.05 *vs.* LPS treatment alone.

Translocation of NF-κB is a hallmark of molecular inflammatory phenomenon involved in the expression and the production of pro-inflammatory mediators and cytokines [40]. Under normal conditions, the p50/p65 heterodimer is bound to IκBα in the cytoplasm [41]. Upon activation by various stimuli such as TNF-α and LPS, IκBα is phosphorylated and degraded. This allows the translocation of NF-κB to the nucleus to activate target genes by binding to κB sites in their promoters [13]. Therefore, we investigated whether **1** and **2** inhibited the phosphorylation and degradation of IκB-α and suppressed the translocation of p65 into the nucleus. As shown in Figure 6A,B, pre-treatment with **1** and **2** for 12 h, at the indicated concentrations, significantly inhibited LPS-induced phosphorylation and degradation of IκB-α, thereby preventing the translocation of p65 into the nucleus. Moreover, we examined the DNA binding activity of NF-κB in the nuclear extracts from RAW264.7 macrophages stimulated with LPS for 30 min. The results indicated that **1** and **2** dose-dependently inhibited NF-κB binding activity that has been increased by LPS (Figure 6C,D). Taken together, these results suggest that compounds **1** and **2** prevented the NF-κB pathway from activating inflammatory enzymes, mediators, and cytokines.

### 2.5. Effects of HO-1 Expression on the Inhibition of Proinflammatory Mediators, Cytokines, and NF-κB Activity by **1** and **2** in LPS-Stimulated RAW264.7 Macrophages

**Figure 7:**
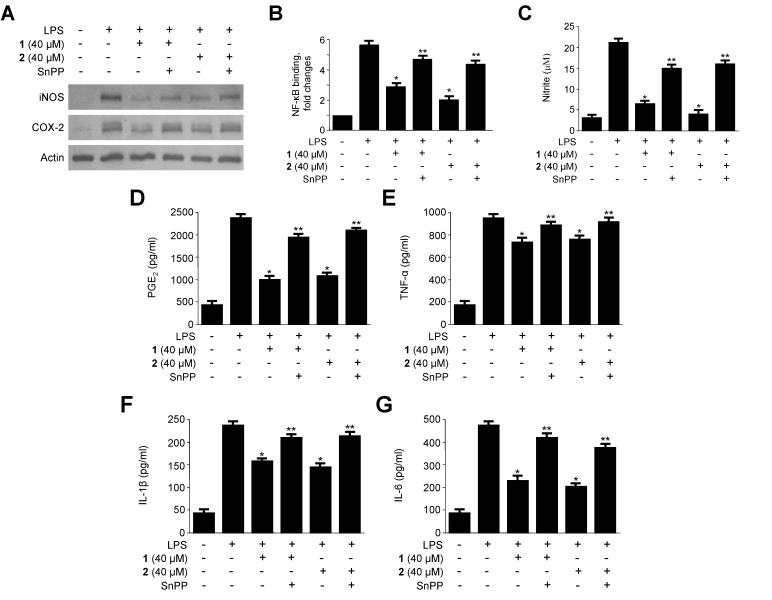
Effects of tin protoporphyrin IX (SnPP) on **1**- and **2**-mediated inhibition of inducible nitric oxide synthase (iNOS) and (cyclooxygenase-2) COX-2 protein expression (**A**); nuclear factor-κB (NF-κB) DNA-binding activity (**B**); nitrite, prostaglandin E_2_ (PGE_2_), tumor necrosis factor-α (TNF-α), interleukin-1β (IL-1β), and interleukin-6 (IL-6) production (**C**–**G**) in RAW264.7 macrophages stimulated with lipopolysaccharide (LPS). Cells were pretreated with **1** and **2** for 12 h in the presence or absence of SnPP (50 μM), and then stimulated with LPS (1 μg/mL) for 18 h (**A** and **C**–**G**) or for 30 min (**B**). Western blot analysis was representative of three independent experiments with similar results. Data are represented as the mean ± S.D. of three independent experiments. * *p* < 0.05 *vs.* LPS treatment alone; ** *p* < 0.05 *vs.* **1** or **2**.

The anti-inflammatory action of HO-1 is mediated by inhibiting the production of pro-inflammatory mediators through NF-κB inactivation [12]. Next, we tested whether compounds **1** and **2**-mediated HO-1 expression was correlated with inhibition of LPS-induced pro-inflammatory mediators. By using tin protoporphyrin (SnPP), a competitive inhibitor of HO activity, we further confirmed the suppressive effects of HO-1 on the production of pro-inflammatory mediators and cytokines as well as on the NF-κB pathway. RAW264.7 macrophages were pre-treated with **1** and **2** (40 μM) for 12 h in the absence or presence of SnPP (50 μM). The inhibitory effects of **1** and **2** toward LPS-stimulated iNOS and COX-2 protein expression, NF-κB binding activity, and production of pro-inflammatory mediators and cytokines including NO, PGE_2_, TNF-α, IL-1β and IL-6 were partially reversed by SnPP (Figure 6). Because SnPP blocks HO enzymatic activity, these data confirmed that **1** and **2** could partially inhibit NF-κB binding activity, NO, PGE_2_, TNF-α, IL-1β and IL-6 production and iNOS, COX-2 protein expression via modulation of HO-1 expression. Therefore, these results suggest that the induction of HO-1 by **1** and **2** is correlated with the ability of these compounds to inhibit the production of pro-inflammatory mediators via the NF-κB pathway (Figure 7). This result suggests that HO-1 expression is involved in the LPS-induced inflammatory cell responses.

## 3. Experimental Section

### 3.1. General

ESIMS data were obtained using a Q-TOF micro LC-MS/MS instrument (Waters, Manchester, UK) located at Korea University, Seoul, Korea. NMR spectra (1D and 2D) were recorded in CDCl_3_ or acetone-*d*_6_ with a JEOL JNM ECP-400 spectrometer operating at 400 MHz for ^1^H and at 100 MHz for ^13^C. Spectra were recorded in CDCl_3_ or acetone-*d*_6_ with resonances at δ_H_/δ_C_ = 7.26/77.2 and δ_H_/δ_C_ = 2.04/29.0. HSQC and HMBC experiments were optimized for ^1^*J*_CH_ = 140 Hz and *^n^J*_CH_ = 8 Hz, respectively. Flash column chromatography was performed using octadecyl-functionalized silica gel C_18_ (12 nm, S-75 μm, YMC, Kyoto, Japan) and silica gel 60 (230–400 mesh, Merck, Darmstadt, Germany). TLC was carded out on silica gel 60 F_254_ plates (Merck). Medium-pressure liquid chromatography (MPLC) was done with a Yamazen MPLC system and an Ultra Pak SI-40A silica gel column (11 mm × 300 mm, Yamazen, Japan). Dulbecco’s modified Eagle’s medium (DMEM), fetal bovine serum (FBS), and other tissue culture reagents were purchased from Gibco BRL Co. (Grand Island, NY, USA). All chemicals were obtained from Sigma Chemical Co. (St. Louis, MO, USA). Antibodies to iNOS, COX-2, phosphor (p)-IκBα, IκBα, p65, PCNA and actin were obtained from Santa Cruz Biotechnology (Santa Cruz, CA, USA), and HO-1, Nrf2 antibodies were obtained from Cell Signaling Technology (Cell Signaling, Danvers, MA, USA). Tin protoporphyrin IX (SnPP), an inhibitor of HO activity, was obtained from Porphyrin Products (Logan, UT, USA). Enzyme-linked immunosorbent assay (ELISA) kits for PGE_2_, TNF-α, IL-1β, and IL-6 were purchased from R&D Systems, Inc. (Minneapolis, MN, USA).

### 3.2. Specimen Collection and Identification of the Marine-Derived Fungus Eurotium sp. SF-5989

*Eurotium* sp. SF-5989 (deposited at the College of Medical and Life Sciences fungal strain repository, Silla University, Busan, Korea) was isolated from an unidentified soft coral that was manually collected using scuba equipment at a depth of 4.5–21 m at Terra Nova bay (74, 37'39.895"S, 164, 14'26.895"E), Antarctica in January 2012. The sample was stored in a sterile plastic bag and transported to the laboratory, where it was kept frozen until further processing. One gram of the sample was ground with a mortar and pestle, and was mixed with sterile seawater (10 mL). A portion (0.1 mL) of the sample was processed utilizing the spread plate method in potato dextrose agar (PDA) medium containing seawater. The plate was incubated at 25 °C for 14 days. After purifying the isolates several times, the final pure culture was maintained as a glycerol suspension (20%, *w*/*v*) at −70 °C. The fungus was identified based on analysis of the ribosomal RNA (rRNA) sequence. A GenBank search with the 28S rRNA gene of SF-5989 (GenBank accession number KF573431) indicated *Eurotium rubrum* (AY004346), *Eurotium repens* (FR839678), *Aspergillus proliferans* (FR848827), *Eurotium chevalieri* (JN938915), *Eurotium amstelodami* (JN938912), *Eurotium herbariorum* (JN938918), and *Eurotium niveoglaucus* (HE578069) as the closest matches, with sequence identities of 100%, 99.48%, 99.48%, 99.48%, 99.48%, 99.48% and 99.35%, respectively. Therefore, the marine-derived fungal strain SF-5989 was characterized as *Eurotium* sp.

### 3.3. Fermentation, Extraction and Isolation from Eurotium sp. SF-5989

The fungal strain was cultured on 50 Petri-dishes (90 mm), each containing 20 mL of potato dextrose agar media (0.4% (*w*/*v*) potato starch, 2% (*w*/*v*) dextrose, 3% (*w*/*v*) NaCl, 1.5% (*w*/*v*) agar). Plates were individually inoculated with 2 mL seed cultures of the fungal strain. Plated cultures were incubated at 25 °C for a period of 14 days. Extraction of the agar media with ethyl acetate (EtOAc) (4 × 1000 mL) provided an organic phase, which was then concentrated *in vacuo* to yield 1.4 g of extract. The EtOAc extract was subjected to C_18_-functionalized silica gel open column chromatography and eluted with a stepwise gradient of 20%, 40%, 60%, 80% and 100% (*v*/*v*) of methyl alcohol (MeOH) in H_2_O (500 mL each). The fraction (1.0 g) eluted with 100% MeOH was subjected to a silica gel column chromatography (3 cm × 19.5 cm) using a gradient elution (*n*-hexane/EtOAc 8:1 to 4:1) to obtain compound **3** (25.2 mg) and nine subfractions (Fractions 52 to 510). The fraction 53 was further purified by silica gel MPLC with a solvent gradient system from 50% *n*-hexane in CH_2_Cl_2_ to 33% *n*-hexane in CH_2_Cl_2_ to afford compounds **1** (1.3 mg, >94% pure in Figure S2) and **2** (2.7 mg, >85.7% pure in Figure S3).

#### 3.3.1. Flavoglaucin (**1**)

^1^H NMR (400 MHz, CDCl_3_) δ: 11.90 (1H, s, 2-OH), 10.23 (1H, s, CHO-7), 6.87 (1H, s, H-4), 5.26 (1H, m, H-2''), 4.33 (1H, br s, 5-OH), 3.27 (2H, d, *J* = 6.96 Hz, H-1''), 2.86 (2H, t, *J* = 7.7 Hz, H-1'), 1.74 (3H, s, H-5'), 1.68 (3H, s, H-4''), 1.56 (2H, m, H-2'), 1.24–1.37 (8H, m, H-3', 4', 5', 6'), 0.88 (3H, t, *J* = 7.0 Hz, H-7'); ^13^C NMR (100 MHz, CDCl_3_) δ: 195.7 (C-7), 156.0 (C-2), 145.1 (C-5), 134.0 (C-3''), 128.7 (C-3), 128.6 (C-6), 125.8 (C-4), 121.3 (C-2''), 117.4 (C-1), 32.1 (C-2'), 31.9 (C-5'), 29.7 (C-3'), 27.1 (C-1''), 26.0 (C-5''), 24.1 (C-1'), 22.8 (C-6'), 18.0 (C-4''), 14.2 (C-7'). HRESIMS: *m*/*z* 303.1963 [M–H]^−^ (calcd for C_19_H_27_O_3_, 303.1960) (Figure S4 and Figure S5).

#### 3.3.2. Isotetrahydro-Auroglaucin (**2**)

^1^H NMR (400 MHz, CDCl_3_) δ: 11.90 (1H, s, 2-OH), 10.22 (1H, s, CHO-7), 6.87 (1H, s, H-4), 5.39 (1H, m, H-5'), 5.27 (1H, m, H-2''), 5.26 (1H, m, H-6'), 4.40 (1H, s, 5-OH), 3.27 (2H, d, *J* = 7.32 Hz, H-1''), 2.86 (2H, t, *J* = 8.4 Hz, H-1'), 2.01 (2H, m, H-2'), 1.74 (3H, s, H-5''), 1.68 (3H, s, H-4''), 1.64 (3H, d, *J* = 4.8 Hz, H-7'); ^13^C NMR (100 MHz, CDCl_3_) δ: 195.6 (C-7), 156.1 (C-2), 145.2 (C-5), 133.9 (C-3''), 133.1 (C-5'), 128.9 (C-3), 128.6 (C-6), 125.9 (C-4), 125.5 (C-6´), 121.4 (C-2''), 117.6 (C-1), 32.4 (C-4'), 31.4 (C-2'), 29.5 (C-3'), 27.2 (C-1''), 25.9 (C-5''), 23.9 (C-1'), 17.8 (C-7'), 17.7 (C-4''). HRESIMS: *m*/*z* 301.1809 [M–H]^−^ (calcd for C_19_H_25_O_3_, 301.1804) (Figure S6 and Figure S7).

### 3.4. Cell Culture and Viability Assay

RAW264.7 macrophages were maintained at a density of 5 × 10^5^ cells/mL in DMEM medium supplemented with 10% heat-inactivated FBS, penicillin G (100 units/mL), streptomycin (100 mg/mL), and l-glutamine (2 mM), and were incubated at 37 °C in a humidified atmosphere containing 5% CO_2_. The effects of various experimental treatments on cell viability were evaluated by determining mitochondrial reductase function with an assay based on the reduction of the tetrazolium salt MTT into fomazan crystals [42]. The formation of formazan is proportional to the number of functional mitochondria in the living cells. For the determination of cell viability, 50 µL of MTT (2.5 mg/mL) was added to cell suspension (1 × 10^5^ cells/mL in each well of the 96-well plates) at a final concentration of 0.5 mg/mL, and the mixture was further incubated for 3–4 h at 37 °C. The formazan that formed was dissolved in acidic 2-propanol, and absorbance was measured at 590 nm. Absorbance of the formazan formed in control (untreated) cells was considered as 100% viability.

### 3.5. Real-Time PCR

Total RNA was isolated from the cells by using Trizol (Invitrogen, Carlsbad, CA, USA), in accordance with the manufacturer’s recommendations, and quantified spectrophotometrically (at 260 nm). Total RNA (1 μg) was reverse transcribed using the High Capacity RNA-to-cDNA kit (Applied Biosystems, Carlsbad, CA, USA). The cDNA was then amplified using the SYBR Premix Ex Taq kit (TaKaRa Bio Inc., Shiga, Japan) by using a StepOnePlus Real-Time PCR system (Applied Biosystems). Briefly, each 20 μL of reaction volume contained 10 μL of SYBR Green PCR Master Mix, 0.8 μM of each primer, and diethyl pyrocarbonate (DEPC)-treated water. The primer sequences were designed using PrimerQuest (Integrated DNA Technologies, Cambridge, MA, USA). The primer sequences were as follows: HO-1, forward 5'-CTCTTGGCTGGCTTCCTT-3', reverse 5'-GGCTCCTTCCTCCTTTCC-3', and GAPDH, forward 5'-ACTTTGGTATCGTGGAAGGACT-3', reverse 5'-GTAGAGGCAGGGATGATGTTCT-3'. The optimum conditions for PCR amplification of the cDNA were established by following the manufacturer’s instructions. The data were analyzed using StepOne software (Applied Biosystems), and the cycle number at the linear amplification threshold (*C*_t_) values for the endogenous control gene (glyceraldehyde 3-phosphate dehydrogenase [GAPDH]) and the target gene were recorded. Relative gene expression (target gene expression normalized to the expression of the endogenous control gene) was calculated using the comparative Ct method (2^−ΔΔ*C*t^).

### 3.6. Western Blot Analysis

RAW264.7 macrophages were harvested and pelleted by centrifugation at 200× *g* for 3 min. Then, the cells were washed with PBS and lysed in 20 mM Tris-HCl buffer (pH 7.4) containing a protease inhibitor mixture (0.1 mM phenylmethanesulfonyl fluoride, 5 mg/mL aprotinin, 5 mg/mL pepstatin A, and 1 mg/mL chymostatin). Protein concentration was determined using a Lowry protein assay kit (Sigma Chemical Co., St. Louis, MO, USA). Thirty microgram of protein from each sample was resolved by 12% sodium dodecyl sulfate-polyacrylamide gel electrophoresis (SDS-PAGE), and then electrophoretically transferred onto a Hybond enhanced chemiluminescence (ECL) nitrocellulose membrane (Bio-Rad, Hercules, CA, USA). The membrane was blocked with 5% skimmed milk and sequentially incubated with the primary antibody (Santa Cruz Biotechnology and Cell Signaling Technology, Santa Cruz, CA, USA) and a horseradish peroxidase-conjugated secondary antibody followed by ECL detection (Amersham Pharmacia Biotech, Piscataway, NJ, USA).

### 3.7. Determination of Nitrite Production and PGE_2_, TNF-α, IL-1β and IL-6 Assays

The production of nitrite, a stable end product of NO oxidation, was used as a measure of iNOS activity. The nitrite present in the conditioned medium was determined using a method based on the Griess reaction [43]. The concentrations of PGE_2_, TNF-α, IL-1β and IL-6 in the culture medium were determined using ELISA kits (R&D Systems) according to the manufacturer’s instructions.

### 3.8. Preparation of Cytosolic and Nuclear Fractions

RAW264.7 macrophages were homogenized in PER-Mammalian Protein Extraction Buffer (1:20, *w*/*v*) (Pierce Biotechnology, Rockford, IL, USA) containing freshly added protease inhibitor cocktail I (EMD Biosciences, San Diego, CA, USA) and 1 mM PMSF. The cytosolic fraction of the cells was prepared by centrifugation at 15,000× *g* for 10 min at 4 °C. Nuclear and cytosolic extracts of cells were prepared using NE-PER^®^ nuclear and cytosolic extraction reagents (Pierce Biotechnology), respectively.

### 3.9. DNA Binding Activity of NF-κB

DNA-binding activity of NF-κB in nuclear extracts was measured using the TransAM kit (Active Motif, Carlsbad, CA, USA) according to the manufacturer’s instructions. Briefly, 30 μL of complete binding buffer (DTT, herring sperm DNA, and binding buffer AM3) was added to each well. The samples were nuclear extracts from RAW264.7 macrophages stimulated for 30 min with LPS and treated with different-concentrations of compounds **1** and **2**. Then, 20 μL of the samples in complete lysis buffer were added to each well (20 μg of nuclear extract diluted in complete lysis buffer). The plates were incubated for 1 h at room temperature with mild agitation (100 rpm on a rocking platform). After washing each well with washing buffer, 100 μL of diluted NF-κB antibody (1:1000 dilution in 1× antibody binding buffer) was added to each well, and then the plates were incubated further for 1 h as before. After washing each well with wash buffer, 100 μL of diluted HRP-conjugated antibody (1:1000 dilution in 1× antibody binding buffer) was added to each well, followed by 1 h incubation as described previously. Then, 100 μL of developing solution was added to each well for 5 min, followed by the addition of stop solution. Finally, the absorbance of each sample at 450 nm was determined with a spectrophotometer within 5 min.

### 3.10. Transfection

The cells were transiently transfected with Nrf2 siRNA for 6 h by using LipofectAMINE 2000™ (Invitrogen), according to the manufacturer’s protocol, and recovered in fresh media containing 10% FBS for 24 h.

### 3.11. Statistical Analysis

Data were expressed as the mean ± S.D. of at least three independent experiments. To compare three or more groups, one-way analysis of variance followed by the Newman-Keuls post hoc test was used. Statistical analysis was performed with GraphPad Prism software, version 3.03 (GraphPad Software Inc., San Diego, CA, USA).

## 4. Conclusions

Naturally occurring compounds that have anti-inflammatory effects may offer a promising strategy to discover and develop drugs for the treatment of some inflammatory diseases. In this study, two benzaldehyde-type fungal metabolites, flavoglaucin (**1**) and isotetrahydro-auroglaucin (**2**) were isolated through bioassay- and ^1^H NMR-guided chemical investigation of the marine-derived fungus *Eurotium* sp. SF-5989 isolated from an unidentified soft coral collected from the Antarctic Ocean. These compounds were shown to inhibit the production of pro-inflammatory mediators, as well as pro-inflammatory cytokines, via inhibition of the NF-κB pathway. Furthermore, the anti-inflammatory effects of flavoglaucin (**1**) and isotetrahydro-auroglaucin (**2**) were shown to be linked to their potential to induce HO-1 expression, which is regulated by Nrf2. The purities of these isolated metabolites were estimated to be less than 95%, however, it was suggested that the presence of impurities would have not influenced the outcome of the presented biological results based on the observations that each compound was presented as a major impurity in the respective congeners, and both compounds showed a similar biological effects in this study. Therefore, these metabolites may be potential therapeutic candidates for the treatment of inflammatory diseases.

## Supplementary Materials

Supplementary figures can be found at: http://www.mdpi.com/1422-0067/15/12/23749/s1.
